# Flood Syndrome: A Rare and Fatal Complication of Umbilical Hernia in Liver Cirrhosis

**DOI:** 10.7759/cureus.9915

**Published:** 2020-08-21

**Authors:** Muhammad Mubbashir Sheikh, Bakhtawer Siraj, Faraeha Fatima, Hamid Ehsan, Muhammad Hassaan Shahid

**Affiliations:** 1 Oncology, Northwestern University Feinberg School of Medicine, Chicago, USA; 2 Internal Medicine, Einstein Medical Center Philadelphia, Philadelphia, USA; 3 Internal Medicine, King Edward Medical University, Lahore, PAK; 4 Internal Medicine, MedStar Union Memorial Hospital, Baltimore, USA; 5 Biomedical Sciences, Georgetown University, Washington, D.C., USA; 6 Pulmonary and Critical Care Medicine, The University of Tennessee Health Science Center, Memphis, USA

**Keywords:** umbilical hernia, ascites, flood syndrome, cirrhosis

## Abstract

Flood syndrome, first reported in 1961 by Frank B Flood, refers to spontaneous umbilical hernia rupture followed by a sudden rush of ascitic fluid. It is a rare sequela in the setting of refractory ascites and liver cirrhosis. Clues to impending rupture include color changes, ulceration, or necrosis over the umbilical hernia that warrants urgent surgical intervention. In this report, we present a unique case of Flood syndrome in a patient with decompensated cirrhosis and umbilical hernia. The patient underwent urgent umbilical herniorrhaphy without mesh; even though adequate postoperative management of ascites was performed, the patient still developed other comorbidities.

## Introduction

Liver cirrhosis is commonly complicated with ascites (>50% of cases) and umbilical hernia (20% of cases) [1]. Spontaneous rupture of umbilical hernia with a resultant sudden rush of ascitic fluid, known as Flood syndrome, is a rare but potentially fatal complication in patients with liver cirrhosis and long-standing ascites [2]. The exact etiology of rupture is largely unknown. The reported factors that explain the mechanism of spontaneous rupture of umbilical hernia in cirrhosis include the inherent weakness of abdominal wall and umbilical vein dilatation and varices formation at the umbilical level secondary to hypoalbuminemia and portal hypertension, respectively, and continuously increased intra-abdominal pressure from ascitic fluid [3-6]. Urgent umbilical herniorrhaphy with primary closure is the preferred intervention in cirrhotic patients presenting with obstructed, incarcerated, or spontaneous rupture of umbilical hernia. However, the postoperative control of ascites is still critical to benefit the repair and prevent the complications and hernia recurrence. We report a rare case of Flood syndrome, which has high mortality and morbidity owing to challenges in medical versus surgical management. This report also highlights that even after recommended interventional steps, a high degree of suspicion should still be maintained to avoid future morbidities associated with Flood syndrome.

## Case presentation

**Figure 1:**
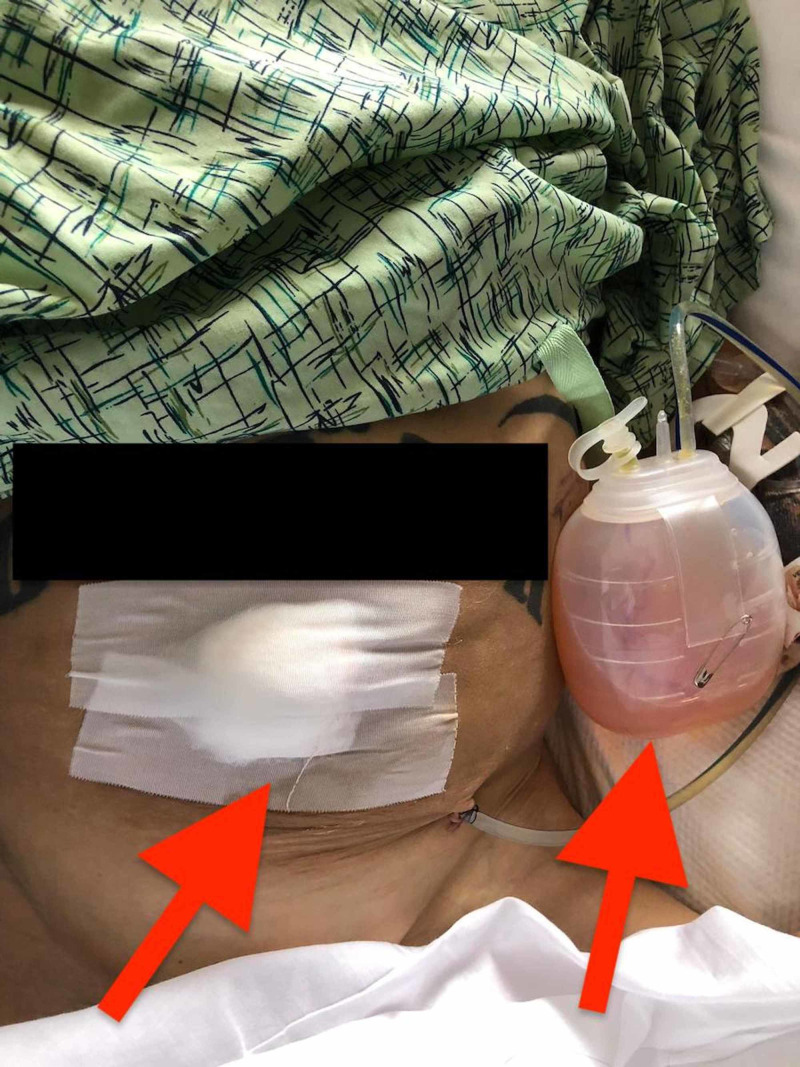
Abdominal dressing status post umbilical herniorrhaphy and intraperitoneal drain for ascitic fluid drainage (red arrows)

A 59-year-old Caucasian male with a past medical history significant for decompensated liver cirrhosis [Child-Pugh grade B, Model for End-Stage Liver Disease (MELD) score of 19] was brought to the hospital by his brother with the chief complaint of worsening cognition and altered mental status that had started about four to five days ago and had gradually worsened since then. Previously, the patient had multiple admissions with hepatic encephalopathy and had been managed successfully with lactulose and rifaximin. In the emergency room, the patient was in no distress and was able to provide most of the history. He was, however, noted to have a waxing and waning level of consciousness. Physical examination revealed normal vital signs except for low blood pressure (90/55 mmHg, mean arterial pressure of 66 mmHg). He appeared cachectic with significant loss of muscle mass. Abdominal examination revealed abdominal distension without any tenderness or guarding, the presence of compressible umbilical hernia (3.5 cm x 3 cm), a shifting dullness, and a fluid wave. The patient was admitted for further workup and underwent diagnostic and therapeutic paracentesis. Analysis of the ascetic fluid did not show any evidence of spontaneous bacterial peritonitis (SBP). On the second day of his hospitalization, his abdominal wall ruptured spontaneously at the umbilical hernia site resulting in ascitic fluid leakage. For prophylaxis against bacterial peritonitis, the patient received broad-spectrum antibiotics, and albumin was provided to maintain intravascular volume. General surgery consultation was obtained, and they decided to take the patient to the operating room (OR) for an urgent hernia repair. Umbilical herniorrhaphy without mesh was performed, and an intraperitoneal drain was placed for abdominal decompression and drainage of ascitic fluid (Figure 1). Postoperatively, his stay was complicated by acute kidney injury (AKI) characterized by an elevated creatinine level of 2.1 from a baseline of 0.6 mg/dl, spontaneous pneumothorax, and failure to thrive. Given the grave prognosis, the patient declined further management and was subsequently discharged on hospice care.

## Discussion

Umbilical hernia accounts for 6-14% of abdominal wall hernias in adults [7]. General risk factors include female sex, obesity, and nulliparity. As compared to the general population, risk factors for the development of umbilical hernia in patients with liver cirrhosis include male sex and ascites, and the prevalence of umbilical hernias in such patients has been estimated at 20% [8]. Cirrhosis in itself being the worst prognostic factor, umbilical hernia in such patients with ascites has a tendency to expand quickly secondary to increased intra-abdominal pressure, and is prone to spontaneous rupture, and consequently, to bowel incarceration, cellulitis, peritonitis, and sepsis [3,5]. Skin ulceration or excoriation almost always precedes the rupture and is usually a warning sign for impending rupture [1].

The prevention of umbilical hernia rupture is dependent on the optimal management of underlying ascites in cirrhotic patients. Conventional strategies include the use of diuretics (furosemide and spironolactone), regular paracentesis, avoidance of alcohol, and non-steroidal inflammatory drugs along with dietary salt and fluid restriction. In cases of failed conservative measures, the surgical options include umbilical herniorrhaphy, transjugular intrahepatic portosystemic shunting (TIPS), peritoneovenous shunting (PVS), and concomitant portal venous decompression [8]. Due to the complexity of syndrome, surgical treatment is not a well-established procedure and is associated with a mortality rate of 0-30%, especially in patients undergoing emergent hernia repair [9-11]. This mortality rate is largely dependent on the patient’s baseline MELD score, albumin level, and platelet count [9-11].

Historically, elective herniorrhaphy has been recommended in cirrhotic patients with well-controlled ascites and no comorbidities [12]. In the presence of complicated hernia or uncontrolled ascites, the procedure is associated with myriad postoperative complications such as wound infection/dehiscence, ascitic fluid leakage, liver failure, and hernia recurrence [1,12]. Presently, urgent umbilical herniorrhaphy without mesh or primary closure is the procedure of choice in cases of rupture with spontaneous ascitic leakage, and it has been shown to reduce the mortality rate to 6-20% [8]. Similarly, our patient was also treated with urgent surgical herniorrhaphy without mesh, and intraperitoneal drain was placed to drain ascitic fluid. However, even after adopting the recommended measures, the patient's postoperative course was still complicated by AKI and other comorbidities. Conversely, studies have also reported instances of reduced hernia recurrence in patients who underwent mesh umbilical herniorrhaphy, but the procedure was still associated with increased risk of wound complications [11,13].

Laparoscopic umbilical herniorrhaphy, a minimally invasive and tension-free procedure, has also been advocated as a beneficial approach in cirrhotic patients with complicated hernia compared to open repair [14,15]. Liver transplantation can be pursued simultaneously with umbilical herniorrhaphy in patients anticipating transplants within three to six months or emergently in patients with hernia complications [16,5]. Lastly, fibrin glue injection can serve as an alternative to surgical options; however, the paucity of data on the procedure limits its applicability [17]. Overall, critical compliance with conservative measures is the only effective strategy at present that can preclude the occurrence of Flood syndrome.

## Conclusions

Flood syndrome is a rare and potentially life-threatening complication in patients with end-stage liver disease and long-standing ascites. The treatment is challenging and not well-defined. A consensus has been reached to follow conservative measures to prevent the occurrence of disease and tailor the surgical options based on the severity of the presentation. Elective umbilical hernia repair has shown favorable outcomes only in patients with intensive preoperative optimization. Urgent herniorrhaphy is necessary for cirrhotic patients with complicated hernias, i.e., obstructed or ruptured umbilical hernia. However, postoperative control of ascites is mandatory to prevent recurrence and further complications.

## References

[1] Choo EK, McElroy S (2008). Spontaneous bowel evisceration in a patient with alcoholic cirrhosis and an umbilical hernia. J Emerg Med.

[2] Flood FB (1961). Spontaneous perforation of the umbilicus in Laennec's cirrhosis with massive ascites. N Engl J Med.

[3] Belghiti J, Durand F (1997). Abdominal wall hernias in the setting of cirrhosis. Semin Liver Dis.

[4] Saleh F, Okrainec A, Cleary SP, Jackson TD (2015). Management of umbilical hernias in patients with ascites: development of a nomogram to predict mortality. Am J Surg.

[5] de Goede B, van Kempen BJ, Polak WG (2013). Umbilical hernia management during liver transplantation. Hernia.

[6] McAlister V (2003). Management of umbilical hernia in patients with advanced liver disease. Liver Transpl.

[7] Shankar DA, Itani KMF, O'Brien WJ, Sanchez VM (2017). Factors associated with long-term outcomes of umbilical hernia repair. JAMA Surg.

[8] Chatzizacharias NA, Bradley JA, Harper S (2015). Successful surgical management of ruptured umbilical hernias in cirrhotic patients. World J Gastroenterol.

[9] Odom SR, Gupta A, Talmor D, Novack V, Sagy I, Evenson AR (2013). Emergency hernia repair in cirrhotic patients with ascites. J Trauma Acute Care Surg.

[10] Gray SH, Vick CC, Graham LA, Finan KR, Neumayer LA, Hawn MT (2008). Umbilical herniorrhapy in cirrhosis: improved outcomes with elective repair. J Gastrointest Surg.

[11] Eker HH, van Ramshorst GH, de Goede B (2011). A prospective study on elective umbilical hernia repair in patients with liver cirrhosis and ascites. Surgery.

[12] Choi SB, Hong KD, Lee JS (2011). Management of umbilical hernia complicated with liver cirrhosis: an advocate of early and elective herniorrhaphy. Dig Liver Dis.

[13] McKay A, Dixon E, Bathe O, Sutherland F (2009). Umbilical hernia repair in the presence of cirrhosis and ascites: results of a survey and review of the literature. Hernia.

[14] Hassan AM, Salama AF, Hamdy H, Elsebae MM, Abdelaziz AM, Elzayat WA (2014). Outcome of sublay mesh repair in non-complicated umbilical hernia with liver cirrhosis and ascites. Int J Surg.

[15] Umemura A, Suto T, Sasaki A, Fujita T, Endo F, Wakabayashi G (2015). Laparoscopic umbilical hernia repair in a cirrhotic patient with a peritoneovenous shunt. Asian J Endosc Surg.

[16] Marsman HA, Heisterkamp J, Halm JA, Tilanus HW, Metselaar HJ, Kazemier G (2007). Management in patients with liver cirrhosis and an umbilical hernia. Surgery.

[17] Sadik KW, Bonatti H, Schmitt T (2008). Injection of fibrin glue for temporary treatment of an ascites leak from a ruptured umbilical hernia in a patient with liver cirrhosis. Surgery.

